# Ethnobotanical study of traditional forage plants in the Gansu–Ningxia–Inner Mongolia junction zone: conservation and sustainable utilization for animal husbandry

**DOI:** 10.1186/s13002-023-00625-0

**Published:** 2023-11-15

**Authors:** Jian Xie, Xiaoqi Liu, Mingxia Luo, Fusong Liu, Sha Liu, Yongxia Zhao, Xingsheng Zhang, Wenji Zhao, Faming Wu

**Affiliations:** 1 School of Pharmacy, Zunyi Medical University, Zunyi, 563000 China; 2 Agricultural and Rural Bureau of Pingchuan District, Baiyin, 730900 China; 3 Sichuan Academy of Grassland Sciences, Chengdu, 611731 China

**Keywords:** Ethnobotany, Forage plants, Sustainable utilization, Conservation, Animal husbandry

## Abstract

**Introduction:**

This study aims to safeguard the ethnobotanical knowledge pertaining to traditional forage plants within the ethnically diverse Gansu–Ningxia–Inner Mongolia junction zone. It seeks to establish a foundation for the sustainable utilization of these traditional resources for animal husbandry.

**Methods:**

A combination of literature research, village interviews, participatory observation, and ethnobotanical quantitative evaluation methods was employed to investigate and study the traditional knowledge of wild forage plants used by local residents in the study area.

**Results:**

Local residents provided information on 73 forage plants, which were identified as 116 distinct wild forage plant species. These plants belong to 22 families and play an active role in the lives of the local inhabitants. Notably, the families Poaceae, Fabaceae, and Asteraceae are prominent, comprising the most abundant and widely utilized wild forage plants. Bing Cao (collectively referring to plants of the Agropyron, Leymus, and Psammochloa), Suo Cao (collectively referring to plants of the genus Stipa), and Ku Cai (encompassing *Lactuca tatarica* (L.) C.A.Mey. and *Ixeris polycephala* Cass.) emerge as the most representative and vital wild forage plants for animal husbandry. Additionally, plants within the Astragalus (referred to collectively as NiaoZi by local residents) in the Fabaceae family, as well as plants from the Amaranthaceae family, exhibit notable significance.

**Conclusion:**

Animal husbandry assumes a pivotal role in the local agricultural economy, and the 116 wild forage plants investigated hold substantial importance in its development. Among these, 59 and 103 plant resources display high developmental potential, making them prospective candidates for high-quality cultivated forage grasses. Additionally, extensive grazing practices have resulted in significant ecological degradation within this already fragile ecosystem. The cultivation of forage grasses and the practice of pen-based animal husbandry may emerge as crucial strategies for sustainable development in this area.

## Background

Animal husbandry holds a paramount position in both traditional and modern agriculture [1]. In the Gansu–Ningxia–Inner Mongolia junction zone, which faces economic challenges, animal husbandry serves as a cornerstone for local social and economic development. This region represents a historical convergence of nomadic and farming ethnic groups in Chinese history, with a diverse coexistence of Han, Mongolian, Hui, and other ethnic minorities. This unique agricultural model combines semi-agricultural and semi-pastoral practices [2, 3].

Forage plants encompass crops, grasses, wild vegetation, directly or indirectly provide nutrition and energy for livestock and poultry. They form the bedrock of livestock farming, while also playing a pivotal role in maintaining ecological balance and safeguarding biodiversity [4].

The Hashan Nature Reserve, located in Jingyuan County, Gansu Province, stands as a rare forest reserve within the Gansu–Ningxia–Inner Mongolia junction zone. Its Mongolian name, “Ha-Si,” meaning “beautiful jade,” reflects its status as a precious gem in the arid expanse of the Loess Plateau. Agricultural production faces severe constraints due to challenging climate conditions, impoverished soil quality, and limited water resources. Consequently, animal husbandry becomes the primary livelihood and source of income for local residents. Animals including sheep, pigs, donkeys, mules, horses, cattle, rabbits, chickens, pigeons, ducks, geese, and even fish from the Yellow River have all contributed to the local economy [5]. Particularly noteworthy are the roles of donkeys, mules, horses, and cattle in traditional agriculture, especially in mountainous cultivation practices [6]. However, with the advent of rural urbanization and agricultural mechanization, the significance of these livestock has dwindled. Once indispensable contributors to agricultural production, animals like donkeys, horses, mules, and other such livestock have become increasingly rare. The remaining livestock primarily serve as a source of meat for human consumption, with even donkeys, which once played a substantial role in regional agriculture, now regarded as a culinary resource. Sheep, including goats, constitutes the predominant livestock in this area, wielding considerable influence over the economic well-being of rural residents. Jingyuan lamb, a renowned delicacy within Gansu Province, is closely associated with this locale.

This multi-ethnic region with its distinct cultural traditions and lifestyle practices harbors rich and diverse traditional knowledge, particularly in the selection, utilization, and management of forage plants. However, with the advancement of societal and environmental changes, this invaluable traditional knowledge is at risk of gradual erosion. Simultaneously, forage plant resources face threats of overexploitation and unsustainable utilization. Hence, a systematic survey, documentation, and analysis of forage plant diversity and associated traditional knowledge in this region bear significant relevance. Such efforts not only serve to protect and perpetuate ethnic cultural heritage but also promote sustainable development of livestock farming, ultimately enhancing the well-being and satisfaction of local residents.

The pursuit of sustainable resource utilization and the establishment of a harmonious balance between agricultural production and ecological preservation have garnered attention from the scientific community [7]. Ethnobotany, as a discipline exploring the relationship between humans and plants, has become an important approach for exploring and preserving traditional knowledge and practices. Internationally, numerous ethnobotanical studies have been conducted on forage plant resources and their utilization in different regions, among diverse ethnic groups, and for various purposes. For example, Sharifian et al. provided a review of global principles in local traditional knowledge regarding forage plant-livestock-herder interactions [8]. Gemedo-Dalle et al. conducted an investigation on plant biodiversity and ethnobotany of Borana pastoralists in southern Oromia, Ethiopia [9]. Fernandez-Gimenez analyzed the ecological knowledge of Mongolian nomadic pastoralists and its relationship with rangeland management [10]. These studies not only provide rich data and information but also demonstrate a variety of research methods and perspectives. However, in China, there is relatively little ethnobotanical research on forage plant resources and their utilization, particularly in the multi-ethnic mixed areas of the northwestern region. There is a lack of systematic, comprehensive, and comparative studies on forage plant resources and their utilization in these areas.

In this context, we employed the ethnobotanical research methodology to comprehensively investigate, document, summarize, and statistically analyze wild forage plant resources and their traditional applications in the multi-ethnic mixed area of the Gansu–Ningxia–Inner Mongolia junction zone. Our study incorporates both quantitative and qualitative analyses. The findings hold significant implications for comprehending the foundational state of forage plant resources in the region, preserving the traditional knowledge system pertaining to their use, and positively contributing to local industrial and plant resource development, ecological preservation, and sustainable agricultural progress.

## Materials and methods

### Research area

**Fig. 1:**
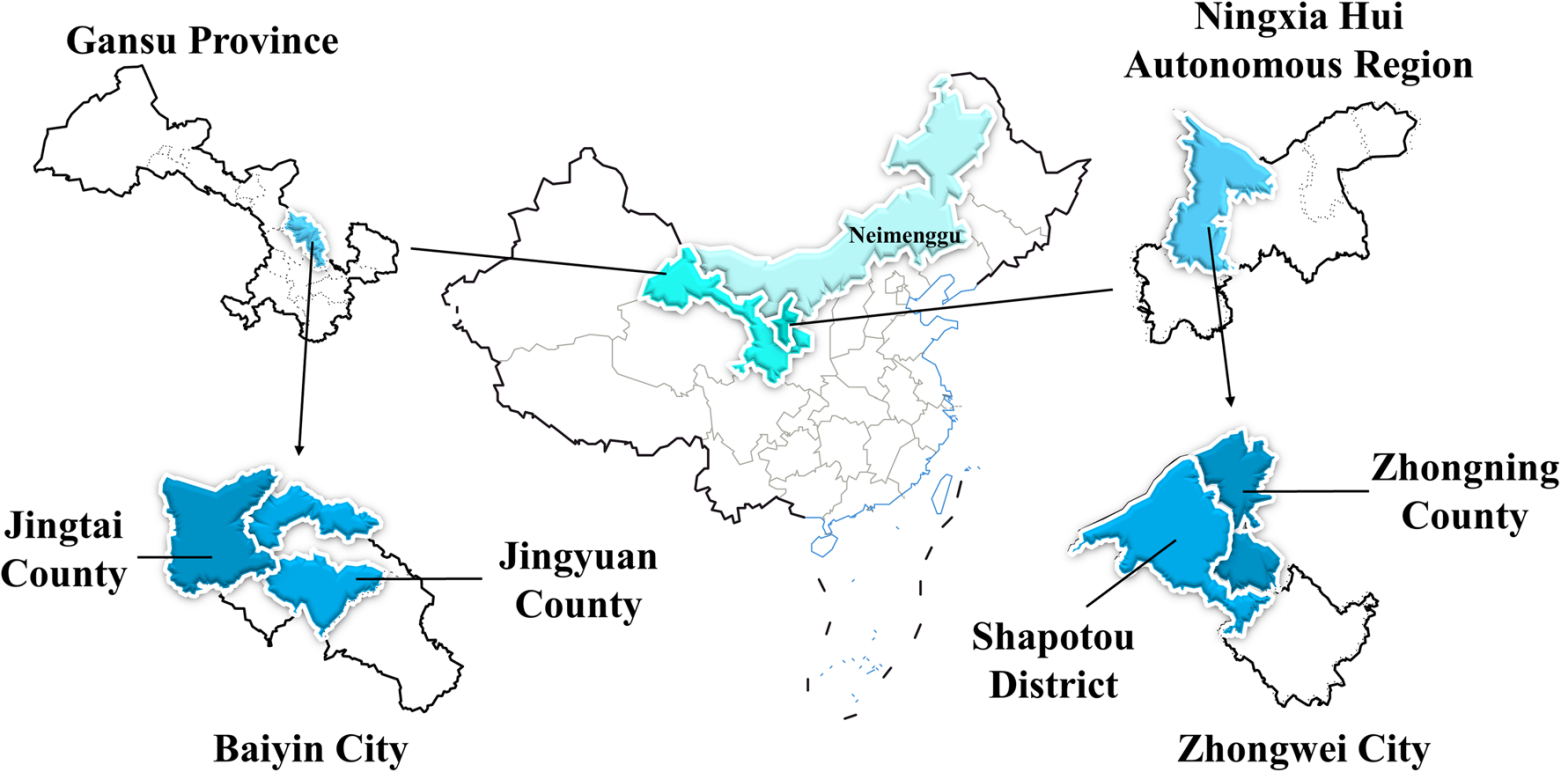
Research Area

**Fig. 2:**
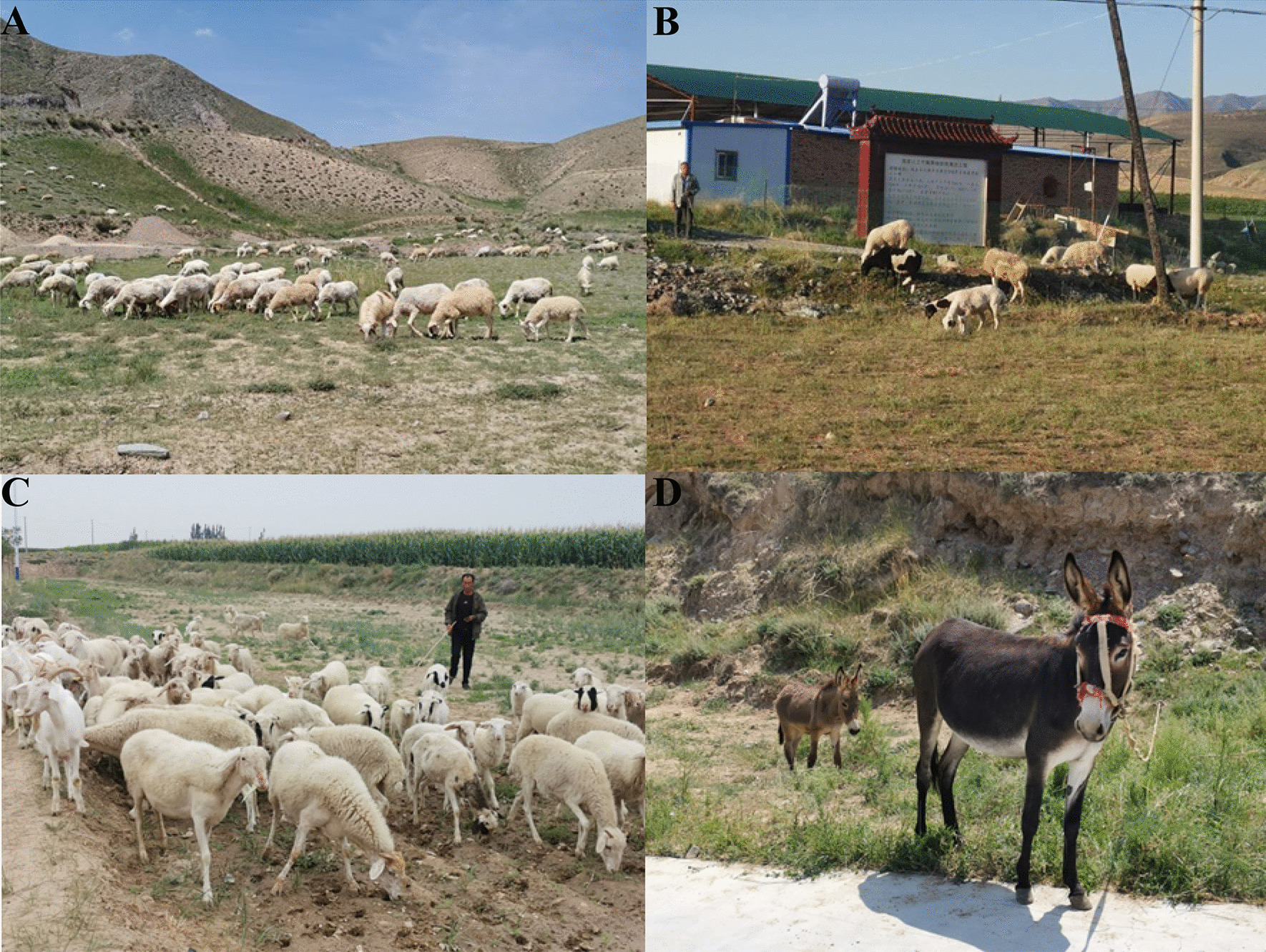
Representative animals raised by local residents (goats, sheep, donkeys)

The study encompasses the eastern region of Baiyin City, including Jingyuan County, Jingtai County, Zhongning County, and Shapotou District, which border Gansu, Ningxia, and Inner Mongolia (Fig. 1). This area spans between 36° N and 37° 50′ N latitude and 103° 33′ E and 106° 7′ E longitude [11]. It lies at the confluence of three significant regions: the Loess Plateau, Inner Mongolia Plateau, and Tengger Desert [12, 13]. The terrain is characterized by hills, mountains, deserts, plains, and terraces, with ravines intersecting and complex wind-sand landforms prevalent [14, 15]. The climate falls within the temperate semi-arid to arid transition zone. Average annual temperatures range from 6 to 9 °C, with evaporation rates between 1829.6 and 3000 mm and annual precipitation ranging from 180 to 450 mm [16–18]. This area serves as a multi-ethnic settlement zone, primarily inhabited by the Han, Hui, and Mongolian ethnic groups. Additionally, Tibetan, Manchu, and other ethnic communities coexist (Table 1) [19]. The enduring amalgamation of these diverse ethnic groups, particularly the perpetual interplay between traditional farming and nomadic cultures, has given rise to a distinctive local agricultural culture that blends elements of both (Figs. 1, 2) [20, 21].

**Table 1:** Basic information of study areas

County	Location	Population	Main ethnic	Main language	GDP/person	Investigation site	Number of Valid Respondents
Jingyuan	E 104° 13′–105° 15′; N 36°–37° 15′	373,000	Han Hui Mongolian Tibetan	Chinese	¥22,410	Shahe Village, Yongxin Township	8
						Hasshan Nature Reserve, Yongxin Township	8
						Shigou Village, Beitang Township	8
Jintai	E 103° 33′–104° 43′; N 36° 43′–37° 38	238,000	Han Hui Mongolian	Chinese	¥26,009	Xindun Village, Caowotan Township	8
						Jinping Village, Wufu Township	8
						Humashui Village, Zhongquan Town	8
Zhongning	E 105° 26′–106° 7′; N 37° 9′–37° 50′	354,400	Han Hui Miao Man	Chinese	¥48,532	Xinbu Village, Xinbu Town	8
						Baima Village, Baima Township	8
Shapotou	E 104° 17′–106° 10′; N 36° 06′–37° 50′	402,000	Han Hui Man Mongolian	Chinese	¥58,807	Jingzhuang Village, Xiangshan Township	8
						Xingren Village, Xingren Town	8

### Basic information of information providers

**Fig. 3:**
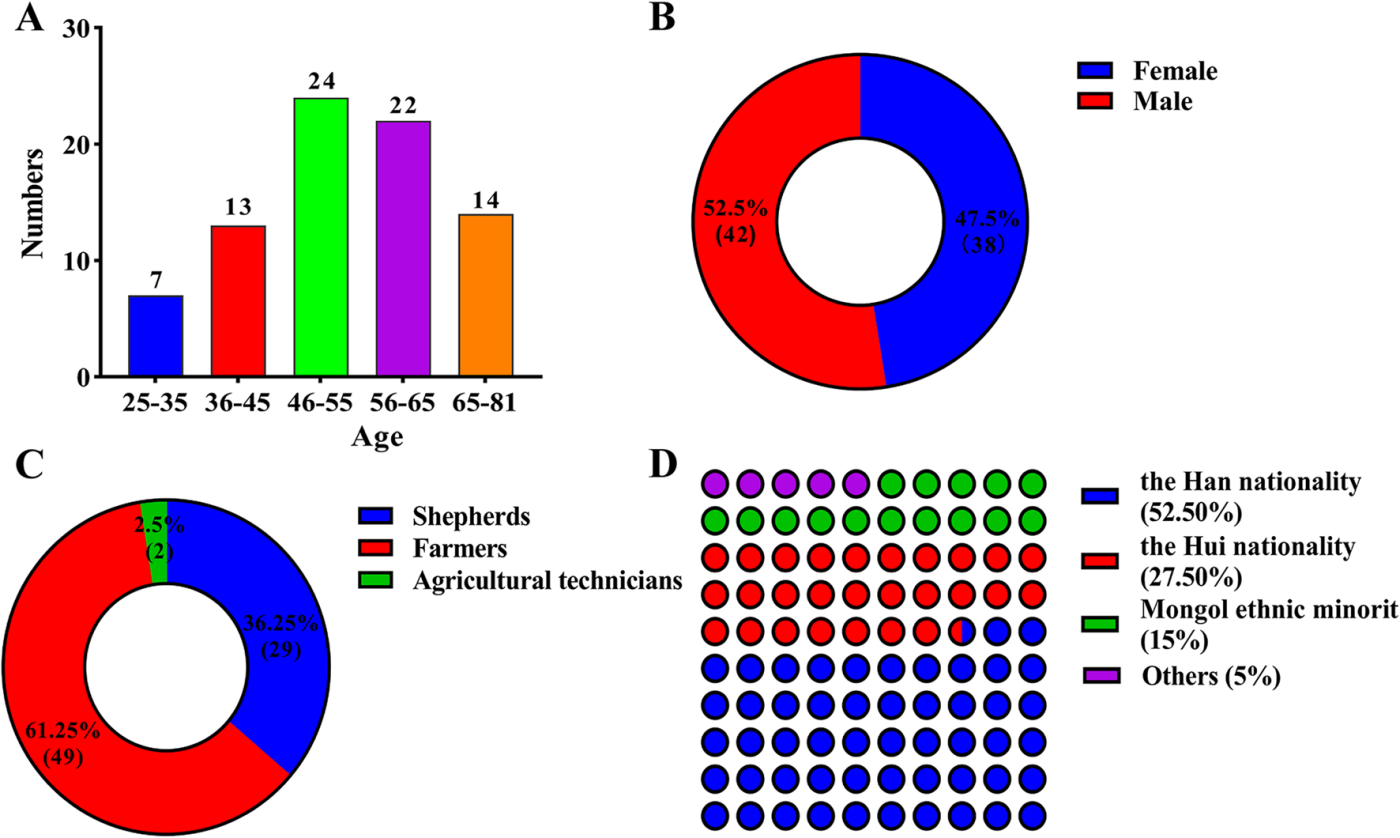
Demographic profile of informants

The study mainly used Mandarin to randomly interview local shepherds and farmers. The information providers were all local rural residents, mainly consisting of shepherds, farmers, and livestock station staff. The 80 information providers had ages ranging from 29 to 81 years old. In particular, 7 of them were in the 25–35 age group, 13 in the 36–45 age group, 24 in the 46–55 age group, 22 in the 56–65 age group, and 14 were older than 65 years old. There were 42 males and 38 females, with a nearly equal gender ratio. The ethnic composition of the providers was 42 Han, 22 Hui, 12 Mongolian, and 4 from other ethnic groups. Among them, there were 29 shepherds (including farmers who had experience in sheep farming), 49 farmers (excluding the 29 shepherds), and 2 agricultural technicians (Fig. 3).

The information providers exhibited distinct characteristics: male, older, and less formally educated individuals offered a wealth of information, which was diverse and valuable. This observation aligns closely with the findings of prior studies on immigrant villages in Ningxia [22]. Interviews with individuals under 25 years old revealed that only a small number possessed knowledge of wild forage plants. As a result, residents under 25 years old were excluded from the group of information providers.

### Ethnobotanical information

The primary informants for this study were local shepherds and farmers. We employed key informant interviews, semi-structured interviews, and participatory rural appraisal methods. The interviews were structured around the “5W + 1H” framework [23], aimed at capturing the traditional knowledge of local residents regarding the use of wild forage plants. Information providers' basic details, as well as local names, utilized parts, processing methods, application techniques, fed animals, feeding seasons, nutritional value, toxicity, and related data for the plants, were recorded, organized, and analyzed.

Participatory observation methodology [24] was applied, involving ten diverse villages. We immersed ourselves in the local milieu, shadowing herdsmen during grazing and farmers during collection. This allowed for an in-depth investigation and research on the primary pastures and breeders in this area, enabling field research on the traditional methods and applications of wild forage plants by local residents. Furthermore, we conducted comparative analyses on the ecological profiles of distinct primary grazing areas and forage cutting zones. Simple quadrat surveys were performed on representative ecological types in this area, with a particular focus on the constituent plant species within each ecological type.

### Evaluation methods

We used Simpson Index (*D*) to evaluate the evenness of forage plant information obtained from different villages: *D* = ∑Pi2; where *D* is the evenness index, *S* is the number of medicinal species, and Pi is the proportion of information providers for medicinal *i* in the total number of information providers for all medicines [25, 26].

Shannon Wiener Index (*H*′), measuring the richness of medicinal information obtained from different villages: *H*′ = − ∑Pi lnPi; where Pi is the probability that the first information provider in village A mentions medicinal *i*, Pi = Ni/*N*, Ni is the number of information providers for medicinal *i* in village A, and *N* is the total number of information providers for all medicines in that village [27].

Sorenson Index (Cs): Cs = 2*j*/(*a* + *b*), measuring the similarity of medicinal information obtained from different villages, where *j* is the number of medicinal species shared by village A and B, and *a* is the total number of medicinal species in village A, *b* is the total number of medicinal species in village B [28].

The Utilization Frequency (HUF), evaluating the adaptation strategy of local people to their surroundings and the utilization degree of medicinal resources in their surroundings: *f* = Nm/Ni; where *f* is The Utilization Frequency; Nm is the number of people who provide information about that medicine; Ni is the total number of information providers [2].

National Cultural Significance Index (NCSI), evaluating the importance of each plant in the lives of local residents: NCSI = FQI × AI × FUI × PUI × MFI × NVI × DSI × 10^–2^; where FQI is frequency of quotation index (the number of people who mention a certain plants among all information providers), AI is availability index, FUI is frequency of utilization index, PUI is parts used index, MFI is multifunctional utilization index, NVI is nutritional value index, DSI is safety index. Refer to “Ethnobotanical Research Methods” [29] to set each index and divide them into grades and assign values.

### Specimen identification

We referred to “Flora of China” full-text electronic version (http://www.iplant.cn/frps) [30], “Illustrated Handbook of Chinese Desert Plants” [31], “Field Identification Manual of Common Plants in China • Qilian Mountain Volume” [32], “Illustrated Handbook of Ningxia Plants,” [33] etc., to identify the plant species collected in the survey, make specimens, sort and analyze various information collected according to research purposes, and draw charts. Related specimens are preserved in Zunyi Medical University Herbarium.

## Results

### Floristic composition of wild forage plants in the region

Local residents furnished information on 73 forage plants by their local names, which we further investigated, identifying 116 distinct wild forage plant species. These 116 plants belong to 21 families, all classified under angiosperms (Table 2). Among these, two families belong to monocotyledons: Poaceae and Liliaceae. Notably, Poaceae is the most prolific family, offering 22 forage grass plants, along with two plants suitable for forage utensils and grasses (*Neotrinia splendens* (Trin.) M.Nobis, P.D.Gudkova & A.Nowak and *Achnatherum caragana* (Trin.) Nevski), and one plant exclusively used for forage utensils (*Achnatherum inebrians* (Hance) Keng). Liliaceae also presents six forage grass plants, though they are relatively scarce. Of these, only two Allium species are widely distributed and also serve as wild vegetables for local residents. Dicotyledons are represented by 19 families and 84 species, prominently featuring Fabaceae and Asteraceae. In particular, we identified 24 wild forage plants in Asteraceae, second only to Poaceae, while Fabaceae presented 15 wild forage plants. Remarkably, Amaranthaceae plants also demonstrated notable performance.

**Table 2:** Inventory of forage plants in the study area

Local name	Species	Family	Used part	Method of use	Main livestock	Feeding season	Toxicity	Nutritional value	Other uses
Bing Cao	*Agropyron cristatum *(L.) Gaertn.	Poaceae	Above-ground	Used for grazing, can be directly used fresh after cutting, or dried for winter reserve feed	Horses, cattle, sheep, donkeys, mules (ruminant animals)	Spring, Summer, Autumn, Winter	Non-toxic	High	Making grass ropes
	*Leymus chinensis *(Trin.) Tzvelev								
	*Leymus secalinus* (Georgi) Tzvelev								
	*Leymus racemosus *(Lam.) Tzvelev								
	*Psammochloa villosa *(Trin.) Bor								
	*Aeluropus littoralis *(Gouan) Parl.								—
	*Calamagrostis epigejos *(L.) Roth								
	*Chloris virgata* Sw.								
Suo Cao	*Aristida adscensionis* L.		Above-ground						
	*Aegilops triuncialis* L.								
	*Stipa caucasica *subsp.* glareosa* (P. A. Smirn.) Tzvelev								
	*Stipa grandis* P. A. Smirn.								
	*Stipa purpurea* Griseb.								
	*Stipa sareptana* A.K.Becker								
	*Stipa tianschanica* Roshev.								
Gu You Zi	*Setaria viridis* (L.) P. Beauv.		Whole Plant	Remove mud and sand from roots after harvesting					Toy making
Xi Ji	*Neotrinia splendens* (Trin.) M. Nobis, P. D. Gudkova & A. Nowak		Tender Leaves/Stems	Grazing		Summer, Autumn		Medium	Making brooms
	*Achnatherum caragana* (Trin.) Nevski			Peeling Stalks for Making Tools		Summer, Autumn			
Zu Ma Zhuang	*Achnatherum inebrians* (Hance) Keng		Stems	Tool making	—	Autumn, Winter	Toxic	—	Making brooms/baskets/backpacks
Lang Wei Ba Cao	*Eragrostis pilosa* (L.) P. Beauv.		Above-ground	Grazed as forage, harvested and used fresh, or air-dried for winter reserve feed	Horses, Cattle, Sheep, Donkeys, Mules	Spring, Summer, Autumn, Winter	Non-toxic	High	—
Xiao Bing Cao	*Eremopyrum orientale *(L.) Jaub. & Spach								
Dao Sheng	*Cenchrus flaccidus* (Griseb.) Morrone								
Xiao Bing Cao	*Phleum pratense* L.								
Lu Wei	*Phragmites australis* (Cav.) Trin. ex Steud.								Weaving Grass Screens
Yan Mai	*Avena sativa* L.		Whole Plant		Feeding mature plants to horses, cattle, sheep, donkeys, mules/Feeding young plants to pigs	Summer, Autumn			—
Ma Ku Cai	*Lactuca tatarica* (L.) C. A. Mey.	Asteraceae	Above-ground	Direct Feeding of Fresh Grass for Horses, Cows, Sheep, Donkeys and Mules	Horses/Cows/Sheep/Donkeys/Mules/Pigs/Rabbits/Chickens/Ducks	Spring, Summer			Wild Vegetables/Medicinal Plants
Hua Ku Cai	*Ixeris polycephala *Cass.								
Tian She Cai	*Sonchus wightianus* DC.								
Leng Hao Zi	*Ajania fruticulosa *(Ledeb.) Poljakov		Dry Branches and Leaves	Winter Grazing	Sheep	Summer, Autumn	Low Toxicity to Bees	Low	Honey Source Plant
Huang Hao	*Artemisia annua *L.						Non-toxic		Medicinal
Hao Cai	*Artemisia frigida* Willd.								Firewood
	*Artemisia stechmanniana* Besser								
	*Artemisia sieversiana *Ehrh. ex Willd.								
Ku Hao	*Artemisia caruifolia* Buch.-Ham. ex Roxb.		—	—	Cow	Autumn, Winter			—
Da Ci Jia Gai	*Carduus crispus *L.		Above-ground	Grazing	Horses/Cows/Sheep/Donkeys	Summer, Autumn, Winter			—
Ci Jia Gai	*Cirsium spicatum* Matsum.							Medium	Medicinal
	*Cirsium arvense* (L.) Scop.								
Ye Ju Hua	*Aster altaicus* Willd.						Low Toxicity to Bees	Low	Honey Source Plant
	*Aster indicus* L.								
Xiao Ku Cai	*actuca tatarica* (L.) C. A. Mey.		Above-›ground	Grazing	Horses/Cows/Sheep/Donkeys/Chicken/Duck	Spring, Summer, Autumn,	Non-toxic	High	Medicinal
	*Crepis rigescens* Diels								
	*Crepidiastrum akagii* (Kitag.) J.W.Zhang & N.Kilian								
Yang Nai Zi	*Takhtajaniantha mongolica* (Maxim.) Zaika, Sukhor. & N. Kilian								
	*Scorzonera sinensis* (Lipsch. & Krasch.) Nakai								
	*Tragopogon capitatus* S.A.Nikitin								
	*Tragopogon kasachstanicus* S. A. Nikitin								
Huang Huang Cai	*Taraxacum scariosum* (Tausch) Kirschner & Štěpánek								Wild Vegetables/Medicinal Plants
	*Taraxacum dissectum* Ledeb.								
	*Taraxacum mongolicum* Hand. Mazz.								
Yu Shu Ye Zi	*Ulmus pumila* L.	Ulmaceae	Leaves/Bark	Grazing	Horses/Cows/Sheep/Donkeys	Summer, Autumn,	Non-toxic	High	Edible
Ma Gan Zi	*Cannabis sativa* L.	Moraceae	branches	Grazing	Horses/Cows/Sheep/Donkeys/Pig	Winter	Low Toxicity	Low	Medicinal
Tie Lian Lian	*Polygonum aviculare* L.	Polygonaceae	Whole Plant	Grazing	Horses/Cows/Sheep/Donkeys	Summer, Autumn,	Non-toxic	Medium	Medicinal
Zhu Ya Liao	*Bistorta vivipara* (L.) Gray		Above-ground	Grazing		Spring, Summer, Autumn,			
Dai Huang	*Rheum rhabarbarum* L.		Roots	捣碎拌入饲料		Spring, Summer, Autumn, Winter			Medicinal
Ye Dai Huang	*Rumex acetosa* L.		Roots						
Hui Tiao	*Chenopodium album* L.	Amaranthaceae							—
Ye Hui Tiao	*Atriplex sibirica* L.		Whole Plant	Grazing		Summer,			—
Li	*Chenopodium album* L.		Whole Plant	Grazing		Summer, Autumn, Winter			Wild Vegetables
Mian Peng	*Corispermum hyssopifolium* L.		Whole Plant			Summer, Autumn, Winter	Non-toxic		—
Tie Sao Zou	*Bassia scoparia* (L.) A. J. Scott		Above-ground	Grazing		Autumn, Winter			Making brooms
Ci Peng	*Kali collinum* (Pall.) Akhani & Roalson		Whole Plant						—
Shui Peng	*Suaeda glauca* (Bunge) Bunge		Whole Plant	Grazing		Winter			—
Fan Zhi Xian	*Amaranthus retroflexus* L.		Whole Plant	Grazing		Summer, Autumn,			—
Yang Liao Zi	*Clematis tangutica* (Maxim.) Korsh.	Ranunculaceae	Above-ground	Grazing	Sheep	Summer, Autumn, Winter	Low Toxicity		Medicinal
	*Clematis fruticosa* Turcz.								
	*Clematis hexapetala* Pall.								
	*Clematis intricata* Bunge								
	*Clematis orientalis* L.								
	*Clematis nannophylla* Maxim.								
Xing Shu	*Prunus armeniaca* L.	Rosaceae	Leaves/Fruits		Horses/Cows/Sheep/Donkeys/Chicken/Duck	Summer, Autumn,	Non-toxic	Medium	Edible/Medicinal
	*Argentina anserina* (L.) Rydb.		Above-ground	Grazing	Horses/Cows/Sheep/Donkeys/Pig	Summer, Autumn,		High	Edible
	*Sibbaldianthe bifurca* (L.) Kurtto & T.Erikss.								—
Huai Shu	*Robinia pseudoacacia* L.	Fabaceae	Leaves/new shoots	Grazing				Medium	Edible/Medicinal
Niao Zi	*Astragalus dilutus* Bunge		Roots	Grazing	Horses/Cows/Sheep/Donkeys/Pig	Autumn, Winter		High	—
	*Astragalus galactites* Pall.								
	*Astragalus steinbergianus* Sumnev.		Above-ground	Grazing	Horses/Cows/Sheep/Donkeys/Pig	Summer, Autumn,			
	*Astragalus scaberrimus* Bunge								
Ning Tiao Zi	*Caragana korshinskii* Kom.		New shoots					Medium	Making tools
Mao Ci	*Caragana pleiophylla* (Regel) Pojark.		New shoots						
Gan Cao	*Glycyrrhiza uralensis* Fisch. ex DC.		Leaves						Medicinal
Ji Guan Hua	*Hedysarum gmelinii* Ledeb.		Above-ground						—
	*Corethrodendron multijugum* (Maxim.) B.H.Choi & H.Ohashi		Above-ground						
Xing An Hu Zhi Zi	*Lespedeza davurica* (Laxm.) Schindl.		Above-ground						
Tian Lan Mu Xu	*Medicago lupulina* L.		Whole Plant					High	
Mu Xu	*Medicago sativa* L.		Above-ground	Grazing	Horses/Cows/Sheep/Donkeys/Pig	Summer, Autumn, Winter	Low Toxicity	High	Edible
Ye Mu Xu	*Melilotus albus* Medic.		Above-ground		Horses/Cows/Sheep/Donkeys		Non-toxic	Medium	—
	*Melilotus officinalis* (L.) Lam.		Above-ground						
Lao Tang Miao	*Erodium stephanianum Willd.*	Geraniaceae	Whole Plant	Grazing		Summer, Autumn,			Edible
	*Geranium dahuricum* DC.		Whole Plant						
Bai Ci	*Nitraria tangutorum* Bobrov	Nitrariaceae	New shoots	Grazing		Summer, Autumn, Winter			
Luo Tuo Peng	*Peganum harmala* L.		Above-ground	Grazing		Autumn, Winter	Low Toxicity	Low	Medicinal
	*Peganum multisectum* (Maxim.) Bobrov								
Sha Zao Shu	*Elaeagnus angustifolia* L.	Elaeagnaceae	Leaves	Grazing			Non-toxic	Medium	Edible
Sha Ji	*Hippophae rhamnoides* L.		Leaves/New Shoots						Edible/Medicinal
Xiao Chai Hu	*Bupleurum smithii* H.Wolff	Apiaceae	Above-ground			Summer, Autumn,		High	Medicinal
Shao Gua	*Cynanchum thesioides* (Freyn) K. Schum.	Apocynaceae	Above-ground	Grazing					Edible
Ku Zi Man	*Calystegia hederacea* Wall.	Convolvulaceae	Whole Plant		Chicken/Duck/Pig			Medium	—
	*Convolvulus arvensis* L.								
Sui Ku Zi Man	*Convolvulus ammannii* Desr.				Horses/Cows/Sheep/Donkeys				
Ma Ya Ci	*Convolvulus tragacanthoides* Turcz.		Above-ground	Grazing		Winter		Low	Firewood
Xiang Ying	*Elsholtzia densa* Benth.	Lamiaceae	Whole Plant	Grazing		Autumn, Winter		Low	Medicinal
E Shui Guan Guan	*Dracocephalum heterophyllum* Benth.			Grazing		Summer, Autumn,		High	—
Gou Qi	*Lycium chinense* Mill.	Solanaceae	Leaves/New Shoots	Grazing		Summer, Autumn, Winter		Medium	Edible/Medicinal
Hei Gou Qi	*Lycium ruthenicum* Murray								
Ye Xi Hong Shi	*Solanum villosum* Mill.		Whole Plant						Edible
	*Solanum torvum* Sw.								
Che Qian Cao	*Plantago asiatica* L.	Plantaginaceae			Horses/Cows/Sheep/Donkeys/Pigs/Rabbits/Chickens/Ducks			High	Medicinal
	*Plantago depressa* Willd.								
Sha Shen	*Adenophora ningxianica* S.Ge & D.Y.Hong	Campanulaceae	Above-ground		Horses/Cows/Sheep/Donkeys				Edible/Medicinal
Sha Cong	*Allium bidentatum* Fisch. ex Prokh. & Ikonn.-Gal.	Amaryllidaceae			Horses/Cows/Sheep/Donkeys/Pigs/Rabbits/Chickens/Ducks				Edible
Ye Jiu Cai	*Allium ramosum* L.								
Men Dong	*Asparagus dauricus* Fisch. ex Link	Asparagaceae			Horses/Cows/Sheep/Donkeys				Medicinal
	*Asparagus gobicus* N.A.Ivanova ex Grubov								
	*Asparagus breslerianus* Schult. & Schult.f.								
Shan Dan Dan	*Lilium pumilum* Redouté	Liliaceae							Edible/Medicinal

Although we encountered monocotyledonous plants in the area, we regrettably did not gather any information regarding their use as forage grasses. Examples include Ephedraceae plants and Pinus plants. It is possible that the scent of these plants correlates with their unpopularity among animals. Additionally, we noted significant variation in the information regarding wild forage grasses provided by informants from different villages. This discrepancy appears directly linked to their respective environments, particularly in areas where they graze or collect forage grasses daily.

### Ecological types and distribution of pastures

**Fig. 4:**
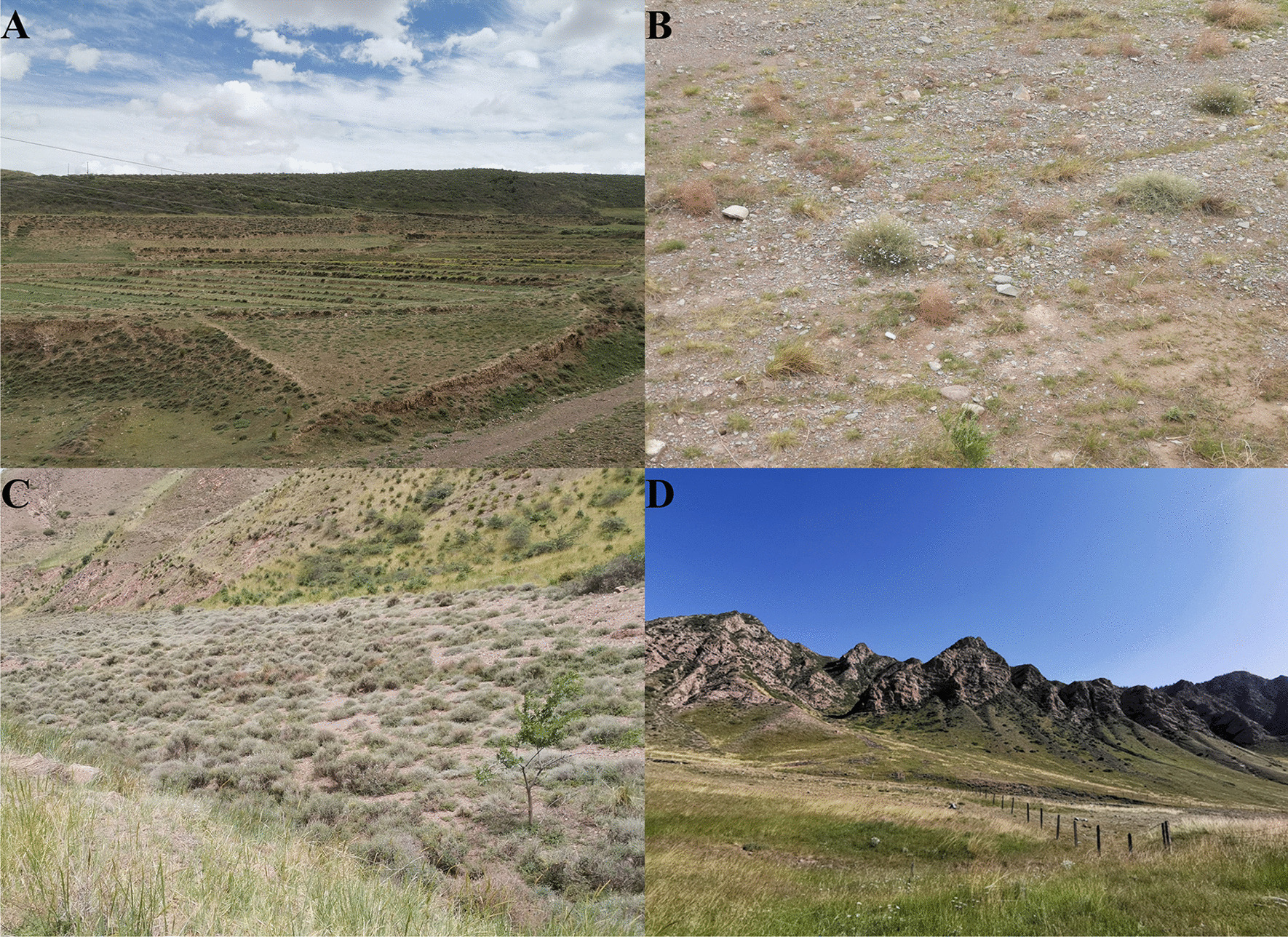
Different ecological types of pastures. **A** Farmland-based Pasture; **B** Desert Pasture; **C** Dry Riverbed Pasture; **D** Mountainous Pasture

The pastures in this area are predominantly comprises four types: farmland pastures, desert pastures, dry riverbed pastures, and mountain pastures. Farmland pastures rely on natural precipitation or irrigation from the Yellow River, supporting the growth of grasses from the Poaceae family and leguminous plants (Fig. 4A). Desert pastures predominantly feature plants from the Asteraceae family, such as Artemisia and *Aster altaicus* Willd (Fig. 4B). Dry riverbed pastures are characterized by species from the *Stipa* genus (needlegrasses) and leguminous plants from the *Astragalus* genus (Fig. 4C). Mountain pastures harbor vegetation primarily composed of *Bistorta vivipara* (L.) Delarbre and *Juniperus procumbens* (Siebold ex Endl.) Miq (Fig. 4D).

Among these, primary forest land, high mountain gravel beach, and high mountain meadow are specialized types limited to specific areas, such as the Hashan area in Jingyuan County and Shoulu Mountain area in Jingtai County, both high-altitude regions within this area. This unique geographical context creates distinctive ecological environments. The majority of this area's ecology falls under the two subtypes of farmland type: afforestation type and sandy river type. Grazing and forage harvesting predominantly occur in mountain type, artificial forest type, desert type, and low mountain meadow type. Due to drought and the expansion of the yellow irrigation area, extensive portions of mountains have been abandoned as high-quality pastures. Furthermore, while low mountain meadow represents the most favorable pasture, this ecological type is notably limited.

### Usage of forage plants in the region

Forage plants utilized by local residents in this region can be categorized into three types based on their uses: single-use (exclusively for forage), dual-use (both medicinal and edible), and auxiliary materials (employed in tool production). The edible category is the most predominant, further subdivided into broad-spectrum forage suitable for all domestic animals, forage specific to ruminant animals primarily for horses, cattle, and sheep, and specialized forage like Ranunculaceae plants of the Clematis genus used exclusively for sheep. Some forage plants, such as *Peganum harmala* L., require frost-induced dormancy before they can be used as forage.

Medicinal usage of forage plants includes species such as *Rheum rhabarbarum* L. and *Rumex acetosa* L. from the Polygonaceae family, which are mainly employed for treating animal ailments. *Cannabis sativa* L. seed oil is also commonly used as an animal remedy for grass knot. Additionally, certain forage plants, such as *Taraxacum mongolicum* Hand-Mazz., *Artemisia annua* L., *B. vivipara* (L.) Delarbre, and *Lilium pumilum* Redouté, are traditional herbal medicines frequently used by local residents.

**Fig. 5:**
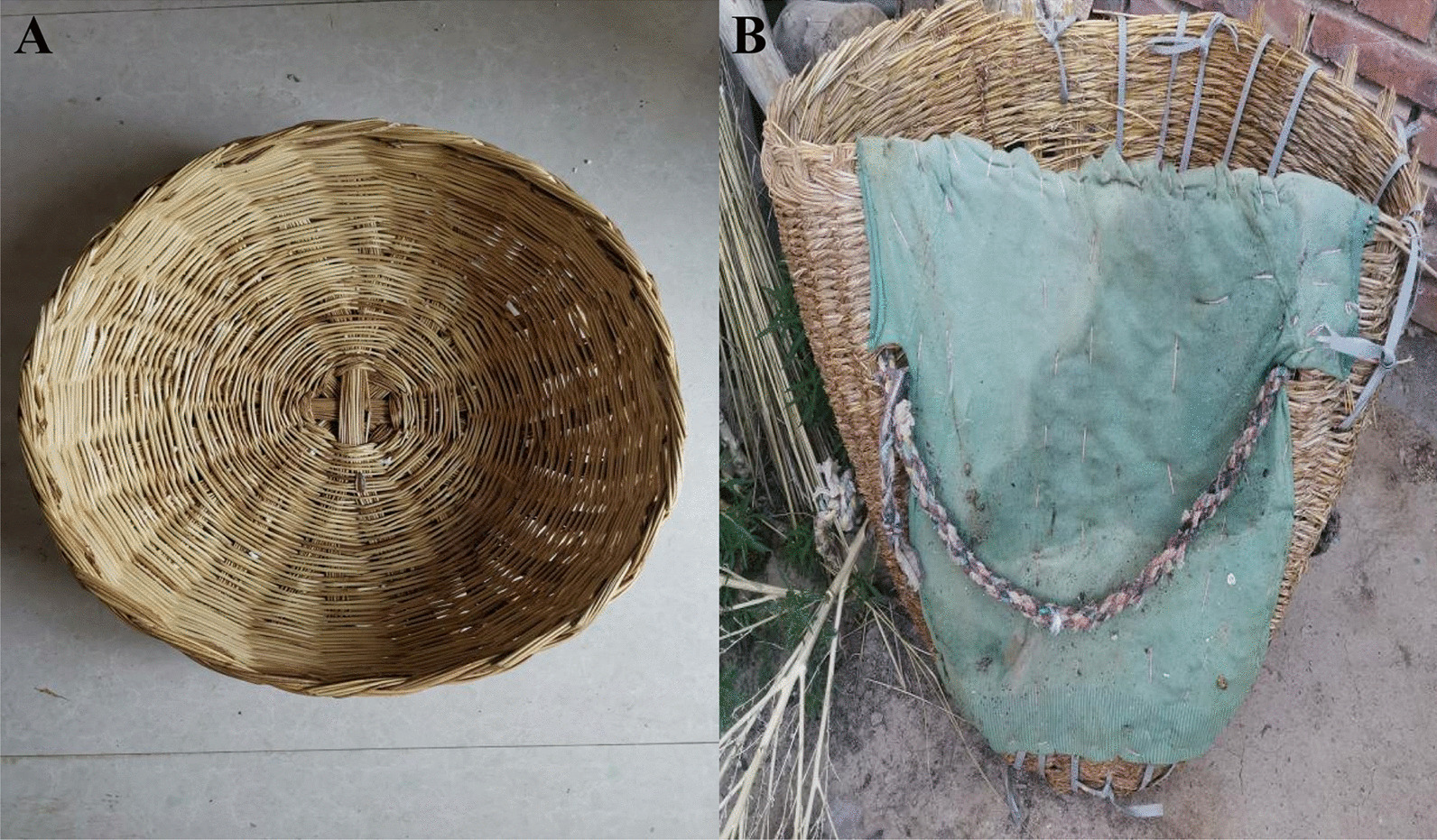
Baskets and backpack made of *Achnatherum splendens* (Trin.) Nevski stems. **A** Basket (used for feeding animals with forage); **B** Backpack (used for harvesting wild forage in the field)

Auxiliary forage plants encompass Timouria (utilized in crafting baskets, brooms, etc.) (Fig. 5A), Agropyron, Leymus, and Psammochloa (with roots used in making grass ropes), as well as Caragana plants of the Fabaceae family (strips employed in weaving baskets, etc.) (Fig. 5B).

In the region, out of the 116 identified wild forage plants, the majority are herbaceous, with 83 being perennial herbs, accounting for 71.55% of the total. Annual herbs are fewer than perennials, with 20 species making up 17.24% of the total. Shrubs comprise 9 species, accounting for 7.76%, while trees contribute only 4 species, amounting to 3.45%. Distinguishing between cultivated and wild trees proves challenging. In terms of utilized parts, perennial herbs mainly consist of above-ground parts, with roots playing a minor role. Annual herbs are mostly used in their entirety, while shrubs and trees are predominantly harvested for leaves, young stems, and branches. Trees, especially, provide a crop of fallen leaves in autumn. However, it is strictly prohibited to gnaw on bark in this area.

In terms of dietary preferences, larger animals like sheep enjoy a wide variety of forage options and can adapt to nearly any plant suitable for forage. Conversely, smaller animals like pigs and poultry have significantly narrower choices. Besides a few types of fresh forage, which can serve as supplements, most wild forage plants are dried and crushed for feed. Fresh forage typically includes plants with succulent leaves, tender stems, and juicy content, along with certain plant seedlings. The former category comprises mainly Asteraceae plants like *I. polycephala* Cass*.*, *L. tatarica* (L.) C.A.Mey., *T. mongolicum* Hand-Mazz., etc., while the latter includes *Avena fatua* L., *Plantago asiatica* L., *Medicago sativa* L., etc.

In essence, wild forage plants in this region primarily serve for natural grazing of animals. Cutting and collecting primarily cater to large labor-providing animals such as horses, donkeys, mules, and cattle to offer supplementary forage at night during summer and autumn. Another collection practice is observed during field weeding, where local residents identify wild plants suitable for forage. Tender and juicy ones (like *I. polycephala* Cass*.*, *L. tatarica* (L.) C. A. Mey., *T. mongolicum* Hand-Mazz., etc.) are typically chopped and mixed with bran to feed pigs (sometimes directly) or chickens. Other forages are also used as supplementary feed for large animals at night. Generally, there's limited large-scale collection of forage for winter hay storage.

### Quantitative evaluation of local residents’ use of wild forage plants

**Fig. 6:**
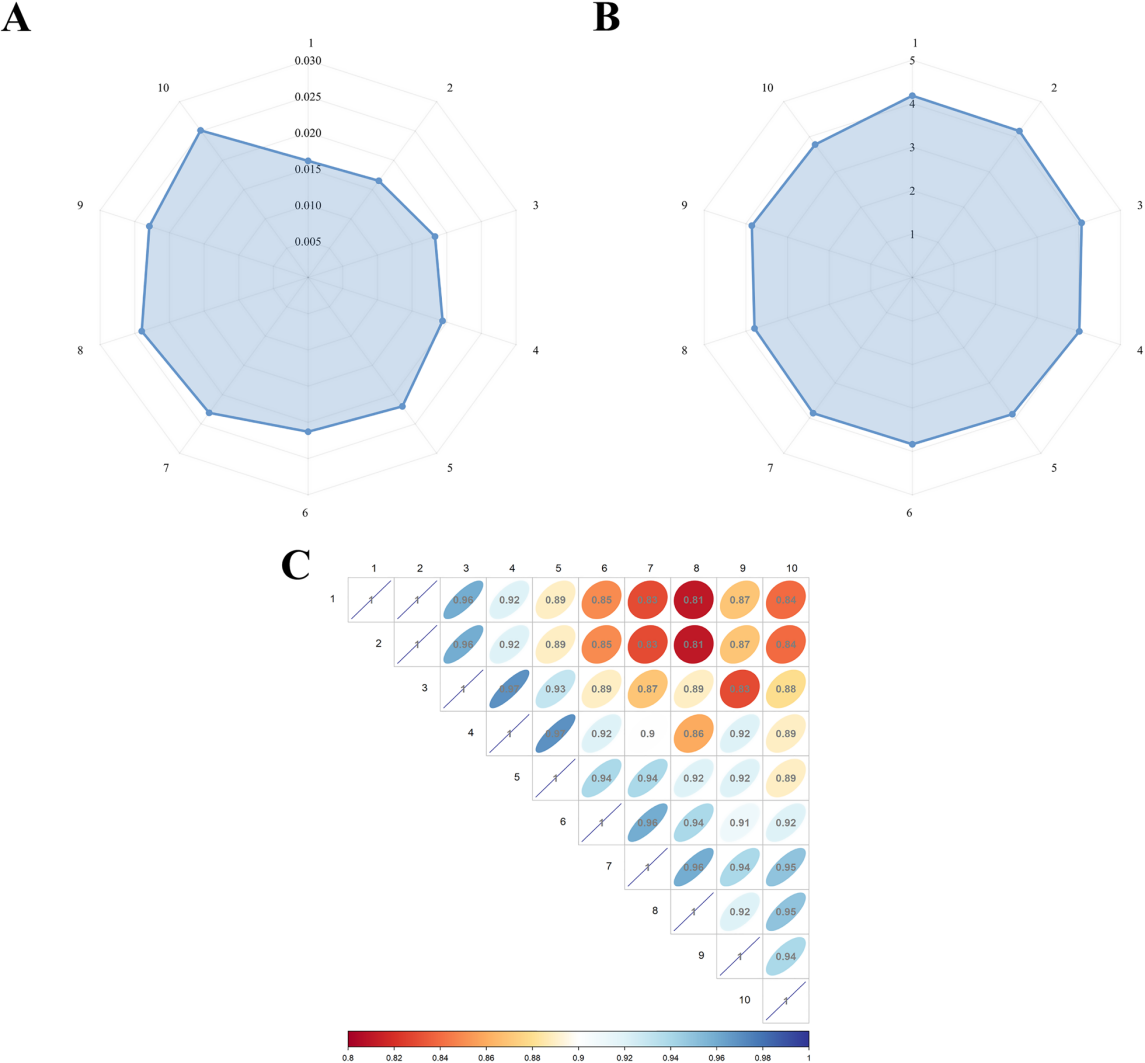
Analysis of Information from Different Villages. **A** Evaluating the Uniformity of Forage Plant Information Obtained from Different Village Surveys Using the Simpson Index. **B** Determining the Richness of Medicinal Plant Information Obtained from Different Village Surveys Using Shannon Wiener Index. **C** Similarity of Medicinal Plant Information Obtained from Different Village Surveys Using Sorenson Index

We conducted a quantitative analysis of the utilization of wild forage plants by local residents, focusing on uniformity, richness, and similarity of the medicinal information gathered from ten surveyed villages (Table 3). The Simpson Index for medicinal information ranged from 0.0161 to 0.0251 (Fig. 6A), while the Shannon Wiener Index ranged between 3.7792 and 4.1815 (Fig. 6B).

**Table 3:** Analysis of evenness and richness in different villages' survey information

Village	1	2	3	4	5	6	7	8	9	10	Total
Simpson Index (*D*)	0.0161	0.0165	0.0183	0.0194	0.0220	0.0213	0.0231	0.0240	0.0229	0.0251	0.0187
Shannon Wiener Index (*H*′)	4.1815	4.1630	4.0681	4.0120	3.8880	3.8365	3.8585	3.7925	3.8579	3.7792	4.0911

Among the surveyed villages, Village 1, known as "Snow Mountain," exhibited the lowest Simpson Index and the highest Shannon Wiener Index. This indicates that the information provided by this village was widely dispersed, with low concentration and uniformity. This observation aligns with our fieldwork, revealing a diverse range of ecological types and abundant plant resources. In contrast, Village 10 (Ningxia) displayed the lowest Shannon Wiener Index and the highest Simpson Index, suggesting concentrated information with minimal variation among providers. It is worth noting that the economic contribution from animal husbandry in this village was relatively modest. Moving forward, we delved into the correlation of information obtained from different villages based on the 73 species of forage plants provided by local residents (Fig. 6C) (Table 4). The Sorenson Index values ranged from 0.81 to 1.00. Broadly speaking, the information acquired from all ten villages exhibited a notable level of consistency. This coherence likely stems from the interplay between plant resource distribution and population migration patterns within the region. We conducted an extensive survey of all 73 species of forage plants in both Villages 1 and 2, which demonstrated the highest degree of similarity. These villages are in close proximity, sharing a highly similar ecological environment. Despite minor distinctions in ecological subtypes, they both present a remarkable convergence in the species composition. In stark contrast, the disparity between Villages 1, 2, and Village 8 was most pronounced. Village 8 is geographically distant from Villages 1 and 2 and, more significantly, exhibits marked differences in ecological types. It is worth noting that our similarity analysis was based on local plant names provided by the residents. Should we employ specific plant species names (totaling 116 species), the dissimilarities between villages would likely be even more pronounced.

**Table 4:** Evaluation of similarity in survey information among different villages

Village	1	2	3	4	5	6	7	8	9	10
1	1.00	1.00	0.96	0.92	0.89	0.85	0.83	0.81	0.87	0.84
2		1.00	0.96	0.92	0.89	0.85	0.83	0.81	0.87	0.84
3			1.00	0.97	0.93	0.89	0.87	0.89	0.83	0.88
4				1.00	0.97	0.92	0.90	0.86	0.92	0.89
5					1.00	0.94	0.94	0.92	0.92	0.89
6						1.00	0.96	0.94	0.91	0.92
7							1.00	0.96	0.94	0.95
8								1.00	0.92	0.95
9									1.00	0.94
10										1.00

These findings underscore that geographical distance and ecological variation play pivotal roles in accounting for inconsistencies in information provided across different villages.

### Assessment of local residents' perception of wild forages

Based on the information provided by the reporters, it is evident that there are notable discrepancies in their assessments of the value (importance) of different wild forages. Consequently, we undertook a quantified evaluation of the significance attributed to 73 species of wild forage plants (classified by local names). This was followed by an assessment of local residents' adaptability to their environment using the Utilization Frequency (HUF), and an evaluation of the importance of wild forage plants in daily life utilizing the National Cultural Significance Index (NCSI).

The Utilization Frequency (HUF) was notably low at 0.06, with *Lycoris squamigera* Maxim. exhibiting the lowest value, closely followed by *Thereianthus spicatus* (L.) G.J.Lewis and *Bupleurum hamiltonii* N.P.Balakr. These plants primarily serve as wild vegetables and medicines in the lives of local residents. It is worth noting that most local residents perceive their use as forage to be an underutilization of these resources. Nine plants obtained a HUF value of 1, indicating unanimous recognition among information providers regarding their pivotal role in the local animal husbandry process. This suggests a comprehensive understanding of these plants among the residents (Table 5).

**Table 5:** Quantitative evaluation index of forage plants in the study area

Local name	FQI	AI	FUI	PUI	MFI	NVI	DSI	NFSI	HUF
Ma Ku Cai	80	4	5	5	3	3	5	3600	1.00
Tian Ku Cai	80	4	5	5	3	3	5	3600	1.00
Hua Ku Cai	67	4	5	4	3	3	5	2412	0.84
Huang Huang Cai	80	4	5	4	3	3	5	2880	1.00
Bing Cao	80	4	5	5	2	3	5	2400	1.00
Gu You Zi	80	4	5	5	2	3	5	2400	1.00
Che Qing Cao	67	4	4	5	2	3	5	1608	0.84
Hui Tiao	80	4	5	4	2	2	5	1280	1.00
Leng Hao Zi	74	4	5	4	2	2	5	1184	0.93
Xi Ji	78	3	5	4	3	2	5	1404	0.98
NiaoZi	44	4	5	5	2	3	5	1320	0.55
Xiao Ku Cai	71	3	5	4	2	3	5	1278	0.89
Yan Mai	78	4	5	5	1	3	5	1170	0.98
Shui Peng	76	4	4	4	2	2	5	973	0.95
Sha Cao	80	4	5	4	1	3	5	960	1.00
Ci Jia Gai	76	3	5	4	2	2	5	912	0.95
Mu Xu	36	4	5	4	2	3	5	864	0.45
Gou Qi	58	4	4	3	2	3	5	835	0.73
Yang Nai Zi	67	2	5	4	2	3	5	804	0.84
Hao Cai	80	4	5	4	2	1	5	640	1.00
Huang Hao	32	4	5	4	3	2	5	768	0.40
Xing Shu	34	4	5	3	3	2	5	612	0.43
Sha Cong	31	4	4	4	2	3	5	595	0.39
Men Dong	31	4	4	4	2	3	5	595	0.39
Yang Liao Zi	56	3	4	4	2	2	5	538	0.70
Ku Zi Man	65	4	4	4	1	2	5	416	0.81
Xiao Bing Cao	55	3	5	4	1	3	5	495	0.69
Ci Peng	65	4	4	4	1	2	5	416	0.81
Xiao Bing Cao	62	2	5	4	1	3	5	372	0.78
Dao Sheng	59	2	5	4	1	3	5	354	0.74
Mian Peng	59	3	5	4	1	2	5	354	0.74
Tie Liang Liang	49	2	4	4	2	2	5	314	0.61
Ye Ju Hua	69	3	5	4	1	2	3	248	0.86
Er Lie Wei Ling Cai	42	3	3	4	1	3	5	227	0.53
Liang Wei Ba Cao	35	2	5	4	1	3	5	210	0.44
Xiang Ying	28	3	3	4	2	2	5	202	0.35
Lao Tang Mao	33	4	3	4	1	2	5	158	0.41
E Shui Guang Guang	43	2	3	4	1	3	5	155	0.54
Ye Hui Tiao	51	3	5	4	1	1	5	153	0.64
Yu Shu Ye Zi	66	3	2	3	1	2	5	119	0.83
Da Ci Jia Gai	19	3	5	4	1	2	5	114	0.24
Ning Tiao Zi	31	3	4	3	2	1	5	112	0.39
Zhu Ya Liao	16	2	4	4	2	2	5	102	0.20
Jue Ma	14	2	3	4	2	3	5	101	0.18
Sha Ci	27	2	2	3	3	2	5	97	0.34
Zhu Ma Zhuang	80	3	5	4	1	1	2	96	1.00
Ye Jiu Cai	5	4	4	4	2	3	5	96	0.06
Tie Shao Zhou	23	2	3	4	3	1	5	83	0.29
Sha Shen	6	2	3	4	3	3	5	65	0.08
Ma Gan Zi	40	2	2	4	2	1	5	64	0.50
Gan Cao	17	3	4	3	2	1	5	61	0.21
Fan Zhi Xian	25	2	3	4	1	2	5	60	0.31
Shao Gua	34	1	2	4	2	2	5	54	0.43
Huai Shu	14	2	2	3	2	3	5	50	0.18
Tian Lan Mu Xu	14	2	3	4	1	3	5	50	0.18
Ye Mu Xu	14	3	3	4	1	2	5	50	0.18
Ji Guan Hua	26	3	3	4	1	1	5	47	0.33
Sandandan	26	1	1	4	3	3	5	47	0.33
Hei Gou Qi	12	2	2	3	2	3	5	43	0.15
Ku Hao	35	2	5	4	1	1	3	42	0.44
Luo Tuo Peng	39	4	3	4	1	1	2	37	0.49
Li	27	2	3	4	1	1	5	32	0.34
Bai Ci	26	2	4	3	1	1	5	31	0.33
Lu Wei	8	1	4	4	2	2	5	26	0.10
Sui Ku Zi Man	15	2	2	4	1	2	5	24	0.19
Ma Ya Ci	20	3	2	4	1	1	5	24	0.25
Xiao Chai Hu	7	1	2	5	2	3	5	21	0.09
Xing An Hu Zhi Zi	14	2	3	4	1	1	5	17	0.18
Da Huang	19	1	2	3	2	1	5	11	0.24
Mao Ci	8	2	3	3	1	1	5	7	0.10
Ye Da Huang	8	1	2	3	2	1	5	5	0.10
Ye Xi Hong Shi	5	2	2	4	1	1	5	4	0.06
Sha Zhao Shu	3	1	1	4	2	2	5	2	0.04

**Fig. 7:**
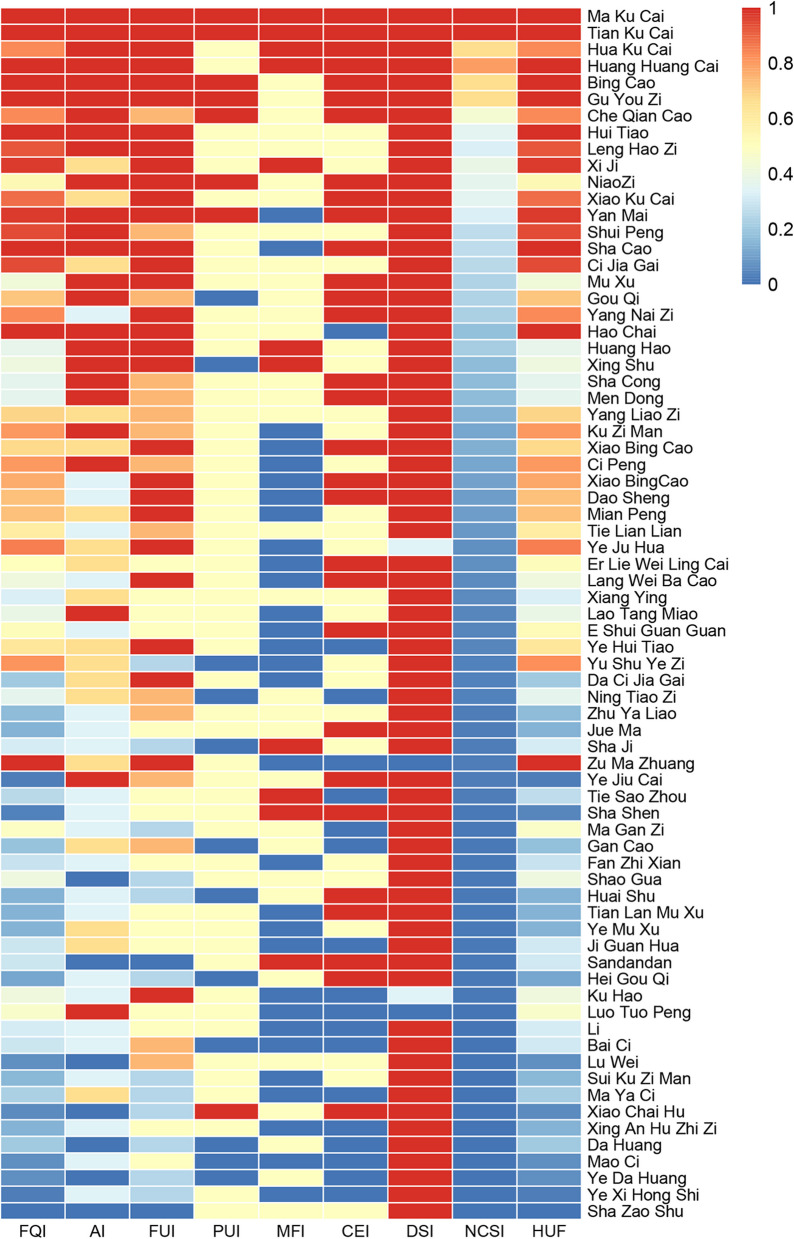
Quantitative evaluation of forage plants

The normalized data from Table 5 are represented in Fig. 7, illustrating the comparative results of the National Cultural Significance Index (NCSI) for wild forage plants in the region. The color transition from blue to red indicates increasing values of the corresponding ordinate. Within the top-tier of importance (NCSI > 1000), 13 plants stood out, all of which are characterized as high-quality forages. Notable representatives include Poaceae Bing Cao (encompassing *Agropyron cristatum* (L.) Gaertn., *Leymus secalinus* (Georgi) Tzvelev, *Elymus dahuricus* Turcz. ex Griseb., *Psammochloa villosa* (Trin.) Bor, etc.), Suo Cao (Stipa plants), Asteraceae’s Ku Cai (*I. polycephala* Cass., *L. tatarica* (L.) C.A.Mey., *Solanum nigrum* L., etc.), Gu Youzi (*Setaria viridis* (L.) P.Beauv.), Yan Mai (*A. fatua* L.), and Fabaceae Niao zi (Astragalus plants). Strikingly, the top four plants in the actual ranking all belong to the Asteraceae. Although their forage use in this area may not be as prominent as Gramineae plants, we observed that these Compositae plants were also the subjects of studies on edible and medicinal plants in this region. This underscores their pivotal role in the lives of local residents, substantiating their higher ranking. In the second tier of significance (1000 > NCSI ≥ 500), there were 12 plants, which are relatively common in this area and serve as prevalent wild forage plants. However, their nutritional value is lower than that of the plants in the first tier. The third tier (500 > NCSI ≥ 100) comprised 19 plants, which were characterized by relatively limited resources and distribution, and may have specific restrictions regarding applicable seasons and animal groups. Lastly, the fourth tier (100 > NCSI) encompassed 29 plants that played a supplementary role as forage plants. These plants had lower resource distribution and nutritional value and were primarily utilized as supplementary forage in instances of forage scarcity during dry or winter seasons.

### Social and economic impacts

**Fig. 8:**
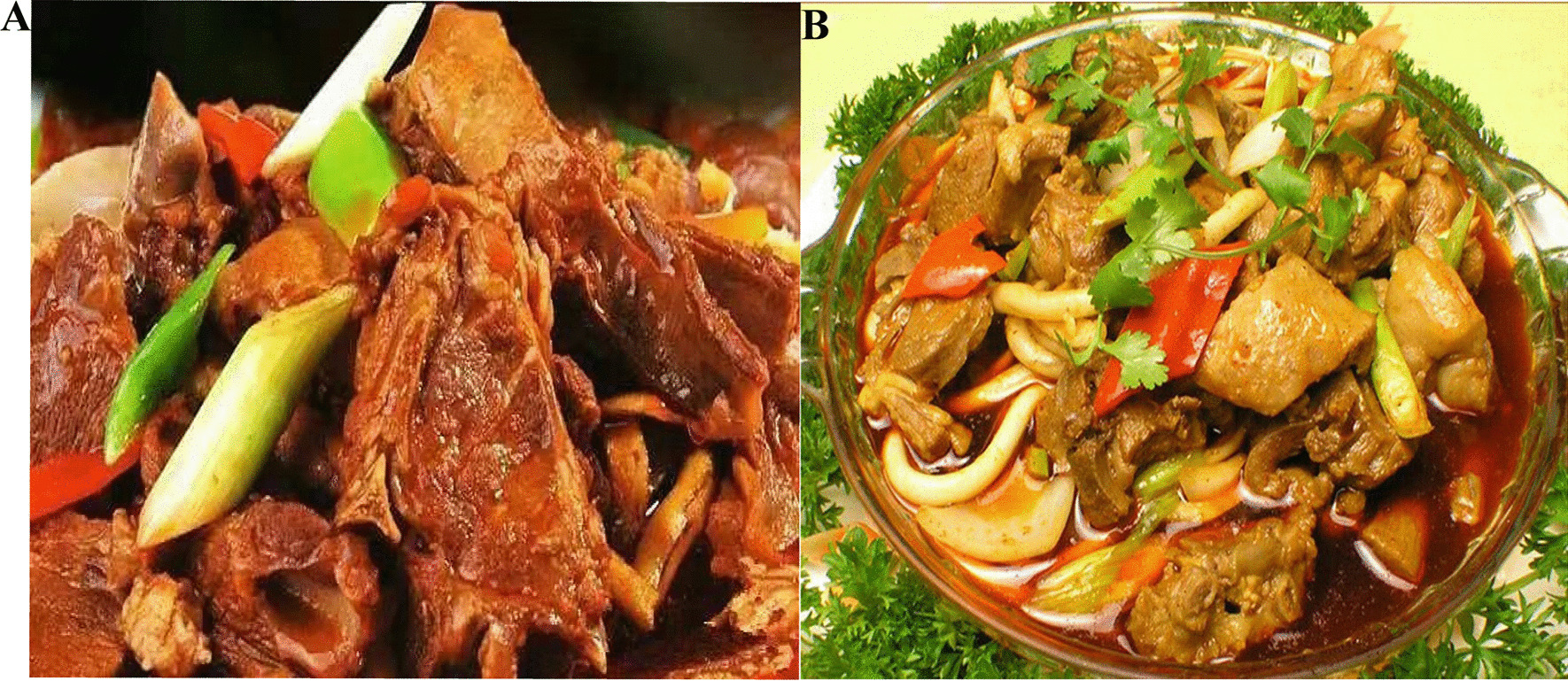
Renowned Brand Products in the Multi-ethnic Inhabited Area at the Junction of Gansu, Ningxia, and Inner Mongolia. **A** Dongwan Donkey Meat, a specialty snack in the Gansu–Ningxia–Inner Mongolia region, known for its unique local flavor and traditional cooking techniques passed down through generations, featuring the distinctive meat texture. **B** Jingyuan Lamb—Utilizing lambs primarily produced from the high-quality local breed of Tan sheep in the Gansu–Ningxia–Inner Mongolia region, Jingyuan Lamb is a culinary creation combining traditional cooking and processing techniques from Jingyuan, Gansu. It is renowned for its tender and succulent meat, not overly fatty, with a delicious and refreshing taste. It has gained widespread popularity and is sold in major cities across the country

We surveyed families engaged in Lycium, Zea and breeding farms in the region to understand the situation of pig, chicken and sheep breeding and sheep herding. Although we did not obtain accurate economic benefit information, most information reporters believed that grazing-type breeding was the agricultural industry with the lowest input cost, highest income and lowest risk in this region. In addition, Jingyuan lamb and Dongwan donkey meat are very famous brand products in this region (Fig. 8). Jingyuan lamb is a national geographical indication product of China (Jingyuan County Government’s slogan: Famous for three thousand miles in Northwest China, attracting guests from all over the world) [34], which played a very important role in promoting the economic development of this region.

## Discussion

The junction area of Gansu–Ningxia–Inner Mongolia is characterized by a dry and rainless climate, with traditional agriculture facing significant challenges due to limited precipitation, especially in areas not directly benefiting from the Yellow River. This has led to a historical reliance on natural rainfall, resulting in a more traditional and less advanced agricultural system in this region [35]. Animal power has played a crucial role in this agricultural system, with large animals being essential for production. While their role in labor has decreased with economic development and government-led initiatives, animal husbandry remains a significant aspect of the local economy. This is evident in the increasing prominence of animals like donkeys and cattle as meat sources, alongside conventional livestock like sheep and pigs. Among these, sheep herding stands out as a vital source of income for local farmers, leading to a comprehensive utilization of plant resources, despite the relatively low species diversity and reserves in the region.

### Characteristics of wild forage plant resources in the region

In terms of wild forage plant resources, our survey identified a total of 116 species in this region. Key families include Poaceae, Fabaceae, Asteraceae, Lamiaceae, and Amaranthaceae. Within these families, certain species, such as Ammophila, Leymus, Stipa, Neotrinia, Astragalus, *Sonchus brachyotus* DC., *L. tatarica* (L.) C.A.Mey., *T. mongolicum* Hand-Mazz., *Chenopodium album* L., and *Chenopodium album* L., were found to be particularly widespread and adaptable for animal forage. Notably, species from the Poaceae and Fabaceae families exhibited marked drought resistance, making them crucial resources for the local animal husbandry industry.

Moreover, we observed a significant presence of wild *M. sativa* L., likely originating from escaped cultivated *M. sativa* L. seeds. This plant assumes paramount importance in the diet of local residents. Additionally, Poaceae's *Oryza sativa* L. and *Avena chinensis* (Fisch. ex Roem. & Schult.) Metzg., together with *riticum aestivum* L., *Zea mays* L., *Panicum miliaceum* L., *Fagopyrum esculentum* Moench, *Pisum sativum* L., and *Lablab purpureus* (L.) Sweet, serve as principal winter animal feed for locals. This prevalence is notably higher than in the southern regions of China, which may underpin the elevated utilization of wild plant resources in this area [36, 37].

Overall, these findings shed light on the intricate relationship between the local environment, agricultural practices, and the utilization of wild forage plants in the Gansu–Ningxia–Inner Mongolia junction area. They underscore the importance of understanding and preserving these resources for sustainable agricultural development in the region.

### Characteristics of wild forage plant resource utilization in the region

The utilization of wild forage plants in the Gansu–Ningxia–Inner Mongolia junction area exhibits distinct patterns, influenced by the specific ecological and agricultural conditions of the region. Poaceae plants are the most extensively utilized forage resources in the region. With the exception of drunken horse grass, which is unsuitable for forage, all other Poaceae plants are used. Species like *E. dahuricus* Turcz. ex Griseb., *A. cristatum* (L.) Gaertn., and Stipa are particularly crucial for livestock farming throughout the year. Local residents have specific names for morphologically similar plants within the Leymus, Elymus, and Agropyron genera, referring to them collectively as "Bing Cao" and needle grass genus plants as “Suo Cao,” despite their lack of relation to true sedge family plants. Additionally, *A. inebrians *(Hance) Keng is highly valued for its stems, which are used in broom-making, a significant local industry.

While the Asteraceae family has a high number of species in the region, their utilization rate is comparatively lower than that of Poaceae plants. There exists a distinct two-level differentiation. Plants like *S. brachyotus* DC., *L. tatarica* (L.) C.A.Mey., *T. mongolicum* Hand-Mazz., *Elaeagnus pungens* Thunb., *S. nigrum* L., and *Crepis rigescens* Diels are considered to have high nutritional value and are widely used in feeding various animals. However, certain Asteraceae plants, such as *Artemisia caruifolia* Buch.-Ham. ex Roxb. and *Inula salsoloides* (Turcz.) Ostenf., cannot be used for forage.

Fabaceae plants, particularly those of the Astragalus genus, serve as important supplementary forage [38]. Their above-ground parts are close to the ground surface and provide limited food resources for livestock. However, their primary value lies in their ability to offer abundant root forage, especially in times of severe drought. These Leguminosae plants are known for their drought resistance and extensive distribution.

Local residents demonstrate a high degree of awareness regarding poisonous plants, likely stemming from their long-term experience. Certain plants from Ranunculaceae, Euphorbiaceae, and Poaceae families, such as *A. inebrians* (Hance) Keng and *Stellera chamaejasme* L., are strictly prohibited for use as forage. This reflects the community's knowledge of potentially harmful plant species. Some forage plants also serve dual purposes as herbal medicines for preventing and treating animal ailments. Examples include *R. rhabarbarum* L. [39], *R. acetosa* L. [40], *Bupleurum smithii* H.Wolff [41] (known locally as *B. hamiltonii* N.P.Balakr.), *A. annua* L. [42], which are used for their medicinal properties.

Overall, the utilization of wild forage plants in the region is intricately tied to the specific needs of the local agricultural practices and the ecological conditions of the area. This comprehensive understanding of plant resources demonstrates the deep knowledge and adaptability of the local community.

### Resource protection and ecology

The rural residents in this region show a very contradictory attitude toward plant resources. On the one hand, they cherish plant resources excessively, and on the other hand, they destroy the natural ecology excessively. This is mainly determined by its dry climate environment. The scarcity of plant resources leads them to develop a value of cherishing and highly utilizing them. The high utilization also brings about serious damage to the ecological environment, especially the damage caused by sheep herding to the ecological environment is particularly prominent in this region. The large-scale ecological *H. rhamnoides* L. forest returned from farmland was eaten up by sheep, and the tree species with scarce species and quantity also became scarcer due to the death caused by cattle, sheep, donkeys, mules and other large animals. The neighborhood disputes caused by livestock gnawing bark are also common.

To address this contradiction, the local government has implemented an enclosure model in recent years. Simultaneously, advancements in modern agriculture and animal husbandry have led to a sharp reduction in the number of large animals, contributing to a gradual improvement in the ecological conditions of the region. Since the 1980s and 1990s, the cultivation of a specialized forage-alfalfa [43] have been introduced in the area. This initiative has played a significant role in alleviating environmental pressure. While many alfalfa fields have aged, they continue to provide crucial support for local animal husbandry. This concerted effort between government policies, modern agricultural practices, and the introduction of specialized forages like alfalfa reflects a proactive approach to balancing resource utilization and environmental preservation in the region. It signifies a recognition of the delicate ecological balance and the necessity to safeguard it for the long-term sustainability of the community.

## Conclusion

This study conducted a comprehensive survey and research on the traditional knowledge of utilizing wild forage plants in the border region of Gansu, Ningxia, and Inner Mongolia. The findings reveal that local residents possess a rich traditional knowledge regarding the use of wild forage plants. These plants serve not only as livestock feed but also play significant roles in folk medicine and handicraft production. Based on a diverse range of wild plant species, local residents have engaged in a diversified farming industry, with sheep husbandry being the predominant livestock sector. In both traditional and modern agriculture, animal husbandry holds a crucial position and has a significant impact on the local socio-economic development. However, with the advancement of socio-economic conditions and environmental changes, these traditional knowledge systems and resources face risks of depletion and overexploitation. Therefore, the preservation and transmission of local residents' traditional knowledge on wild forage plants hold paramount importance. This endeavor contributes to the safeguarding of ethnic cultural heritage, promotion of sustainable development in animal husbandry, and enhancement of the livelihoods and well-being of local residents. Furthermore, this study provides crucial references for understanding the fundamental aspects of wild forage plant resources in this region, preserving the traditional knowledge system regarding the utilization of wild forage plants, and gaining insights into local industrial development, plant resource utilization, ecological conservation, and sustainable agricultural development.

## Data Availability

All data, materials, and information are collected from the study sites.

## Abbreviations

HUF: The Utilization Frequency
NCSI: National Cultural Significance Index
FQI: Frequency of quotation index
AI: Availability index
FUI: Frequency of utilization index
PUI: Parts used index
MFI: Multifunctional use index
NVI: Nutritional value index
DSI: Drug safety index
